# The Top 50 Articles and Authors of the New Millennium in Psychiatry: A Bibliometric Analysis

**DOI:** 10.7759/cureus.54762

**Published:** 2024-02-23

**Authors:** John L Havlik, Sofía I Uranga, Megan S Lee, Seneca Magallanes, Syed Wahid, Taeho (Greg) Rhee

**Affiliations:** 1 Psychiatry, Yale School of Medicine, New Haven, USA; 2 Biological Sciences, University of Chicago, Chicago, USA

**Keywords:** great works in psychiatry, psychiatry leadership, h-index, top authors, bibliometric analysis

## Abstract

The field of psychiatry faces significant challenges in the new millennium, marked by a surge in mental health diagnoses coupled with barriers to accessing adequate care. Despite obstacles, notable advancements have been achieved throughout the field, including the release of DSM-5, the introduction of esketamine, and the development of innovative assessment tools. This study aims to comprehensively analyze recent advances in psychiatry by examining the top 50 most cited articles and authors since 2000, addressing a gap in the literature left by previous subfield-focused bibliometric studies. Utilizing the Web of Science (WOS) database, this bibliometric analysis examined all publications in psychiatric journals from January 1, 2000, to September 18, 2022. The top 50 most cited articles and authors were identified and characterized based on various metrics, including times cited, article type, and institutional affiliations. WOS extracted 699,005 articles, with authors from the United States contributing the highest number of publications. The top 50 articles spanned a variety of formats, with cross-sectional studies, new measures, literature reviews, and randomized controlled trials being the most prevalent. The *American Journal of Psychiatry* emerged as the leading journal, hosting eight of the top 50 articles. Among the top 50 authors, female representation was limited, comprising 24% of first authors and 22% overall. Institutional affiliations revealed a majority of top authors worked at universities affiliated with the top 40 NIH-funded departments of psychiatry, with those affiliated with Harvard University leading in authorship contributions. This study sheds light on recent advancements in psychiatry, emphasizing the underrepresentation of female authors and the prevalence of top authors affiliated with major NIH-funded programs. This bibliometric analysis provides a comprehensive overview of recent advances and the top recent contributors in the field, fostering a deeper understanding of the evolving landscape of psychiatry in the new millennium.

## Introduction and background

The new millennium has brought substantial challenges to the field of psychiatry. Data suggest that over 20% of American adults now experience mental illness; the years between 2008 and 2019 saw a 30% increase in mental health diagnoses in adults over 18 [1-4]. However, the increased need for mental healthcare is not reflective of the number of patients receiving the care they need to safely and properly manage their mental health; social stigma, insurance coverage, and lack of access to skilled psychiatric clinicians continue to impede the provision of psychiatric care in this country [5,6]. The balance between demand and supply of mental healthcare in the United States is a problem yet to be resolved, a problem that requires both scaling of existing best practices and research into new and innovative solutions.

While the challenges to psychiatric care in this country are immense, the new millennium has thus far brought great progress to the field. The release of the DSM-5 in 2013 updated psychiatry’s toolbox to treat and diagnose [7]. The discovery and approval of esketamine, the first novel method of action antidepressant in 50 years, has revolutionized options for those suffering from treatment-resistant depression [8]. The development of the Connor-Davidson Resilience Scale (CD-RISC) was able to provide an insightful predictor for patient improvement in response to treatment [9]. Similarly, the assessment and analysis of the CIDI-SF scale, K10/K6 nonspecific distress scales, and the WHO-DAS for the screening of serious mental illnesses have allowed crosstalk between community and clinical epidemiology [10]. The new millennium has brought psychiatry into an unprecedented era of ground-breaking research.

With both the volume of new research in the field and the great progress that has been made in the past two decades, a survey of the most impactful recent advances and top researchers is needed to inform those new to psychiatry. Many bibliometric studies have been dedicated to aggregating and analyzing top-cited articles in psychiatric sub-fields. However, these studies do not provide a broad survey of recent advances in the field [11-14]. To our knowledge, there exists only one other notable psychiatry-wide bibliometric analysis: in 2013, Mazhari sought to identify characteristics of the top 100 most cited articles published in international journals dedicated to psychiatry [15]. Mazhari’s collected articles were published between 1957 and 2005; a study of recent advances in the field and characterization of those innovators making these advances is needed to inform field-wide discussion.

In this study, we use data from the Web of Science (WOS) to analyze all publications in psychiatric journals from January 1, 2000, to September 18, 2022. We characterize both the top 50 most cited articles and the top 50 most impactful authors in psychiatry since 2000 and categorize relevant metrics for each top paper and author. This bibliometric analysis seeks to bridge the current gap in literature by providing a comprehensive analysis of the top authors and articles cited in the entire field of psychiatry in the new millennium, an evaluation that may prove a valuable window into major recent advances and those making these advances in the field.

## Review

Methodology

Data Source and Study Sample

This study used data from the Web of Science (WOS) database (Clarivate Analytics PLC), a paid-access platform aggregating scientific and medical journal databases commonly used in bibliometric analyses [16]. The WOS Core Collection platform contains over 85 million records from 1900 present, with data on over 1.8 billion citations [17]. In this study, we included the entire sample of papers published 01/01/2000-10/14/2022 that were published in the journal category “Psychiatry.” While results were tabulated as returned in WOS, cross-verification of all articles was performed in PubMed to verify article authorship. Works that were not original research articles, meeting abstracts, research letters, or editorial materials were excluded from our analysis. Importantly, because WOS defines article categories in part based on journal of publication, psychiatry articles published in general medicine journals were excluded from our analysis.

Measures

Author-specific measures included name, times cited, most cited article, year most cited article was published, average number of times most cited article was cited per year, number of publications, H-index, current or most recently affiliated academic institution as determined through Google search, if the author’s primary institution was one of the top 40 departments of psychiatry receiving National Institutes of Health funding in 2022 as per the Blue Ridge Institute for Medical Research, and highest post-graduate degree(s) [18]. Article-specific measures included times cited, article type (cross-sectional study, clinical classification system, literature review, new measure, new diagnostic tool, randomized controlled trial meta-analysis, statistical method, literature review, or new dataset), title, author names, journal, and year.

Analysis

The top 50 most-cited studies in psychiatric journals were characterized in order of times cited as per WOS records, while the top authors were characterized in order of number of total citations. Median times cited and interquartile ranges (IQR) were calculated for the top articles. The 50 top authors in psychiatry of the new millennium were assessed in this analysis as those authors who were 1) either first or senior (last) authors in one of the top 50 studies previously identified and 2) in the top 50 most-cited psychiatrists within this group. Like top articles, these top authors were also characterized in order of overall times cited per WOS. All analysis was performed in Microsoft Excel.

Study population and characteristics

The WOS database returned a total of 699,005 results for articles published in psychiatric journals from 01/01/2000-09/18/2022. The studies included in this analysis were comprised of 54% original articles (n = 376,569), 29% meeting abstracts (n = 200,475), 5% letters (n = 31,927), 13% editorial materials (n = 49,511), and 6% reviews (n = 40,523). The five countries with the largest number of publications were the United States (35.5%, n = 247,915), followed by England (11.5%, n = 80,243), Germany (8.2%, n = 57,174), Australia (6.6%, n = 45,922), and Canada (6.2%, n = 43,074).

Top articles in psychiatric journals

The 50 highest-cited publications in psychiatric journals since 2000 are listed in Table 1. These publications were cited between 15,001 and 1,964 times. “Lifetime prevalence and age-of-onset distributions' of DSM-IV disorders in the national comorbidity survey replication” was the highest-cited publication with 15,001 citations. In this landmark 2005 cross-sectional analysis, Kessler et al. shed light on the prevalences of DSM-IV psychiatric disorders in adults, work that has proven foundational not only to the field of psychiatric epidemiology but to psychiatry as a whole. A commentary letter entitled “Diagnostic and Statistical Manual of Mental Disorders” was reported as the second highest cited publication with 8,592 citations in the WOS database; cross-validation of this article using the article’s PubMed ID (21741095) revealed only 24 citations. Upon further inquiry, it became clear this article had erroneously received credit for citations in WOS that should have been attributed to the DSM-V. Finally, “Short screening scales to monitor population prevalences and trends in non-specific psychological distress” was reported as the third highest-cited article with 5,559 citations in WOS. In this article describing new clinical measures, Kessler et al. discuss the implementation and validation of two screening scales for psychological distress, the K6 and K10.

**Table 1:** The 50 most cited articles of the new millennium in psychiatric journals. RCT: Randomized Controlled Trial. *Times cited based on data from Web of Science, 10/14/2022. **First author, second author, and last author are listed, if applicable, in this column. ***Substantial misattribution of citations of DSM-V to this article in Web of Science.

Table 1. The 50 most cited articles of the new millennium in psychiatric journals.							
Rank	Times cited*	Publication type	Article title (as published)	Type of article	Author last name, first initials**	Journal	Year published
1	15,001	Article	Lifetime prevalence and age-of-onset distributions' of DSM-IV disorders in the national comorbidity survey replication [19]	Cross-sectional	Kessler, RC; Berglund, P; Walters, EE	Archives of General Psychiatry	2005
2***	8,592	Letter	Diagnostic and statistical manual of mental disorders [20]	Commentary	Mittal, VA; Walker, EF	Psychiatry Research	2011
3	5,559	Article	Short screening scales to monitor population prevalences and trends in non-specific psychological distress [21]	New measure	Kessler, RC; Andrews, G; Zaslavsky, AM	Psychological Medicine	2002
4	5,184	Review	The validity of the Hospital Anxiety and Depression Scale. An updated literature review [22]	Literature review	Bjelland, I; Dahl, AA; Neckelmann, D	Journal of Psychosomatic Research	2002
5	4,078	Article	Development of a new resilience scale: The Connor-Davidson Resilience Scale (CD-RISC) [23]	New measure	Connor, KM; Davidson, JR	Depression and Anxiety	2003
6	4,033	Review	The endophenotype concept in psychiatry: etymology and strategic intentions [24]	Review	Gottesman, II; Gould, TD	American Journal of Psychiatry	2003
7	4,026	Article	Psychometric properties of the strengths and difficulties questionnaire [25]	Cross-sectional	Goodman, R	Journal of the American Academy of Child and Adolescent Psychiatry	2001
8	3,745	Editorial Material	Research Domain Criteria (RDoC): toward a new classification framework for research on mental disorders [26]	Clnical classification	Insel T; Cuthbert, B; Wang, P	American Journal of Psychiatry	2010
9	3,438	Article	The "Reading the Mind in the Eyes" test revised version: a study with normal adults, and adults with Asperger syndrome or high-functioning autism [27]	New diagnostic tool	Baron-Cohen, S; Wheelwright, S; Plumb, I	Journal of Child Psychology and Psychiatry	2001
10	3,336	Article	Lifetime prevalence of mental disorders in U.S. adolescents: results from the national comorbidity survey replication-adolescent supplement (NCS-A) [28]	Cross-sectional	Merikangas, KR; He, JP; Swendsen, SA	Journal of the American Academy of Child and Adolescent Psychiatry	2010
11	3,240	Article	The worldwide prevalence of ADHD: a systematic review and metaregression analysis [29]	systematic review	Polanczyk, G; de Lima, MS; Rohde, LA	American Journal of Psychiatry	2007
12	3,183	Article	Prevalence, severity, and comorbidity of 12-month DSM-IV disorders in the National Comorbidity Survey Replication [30]	Cross-sectional	Kessler, RC; Chiu, WT; Walters, EE	Archives of General Psychiatry	2005
13	3,175	Review	Neurocircuitry of addiction [31]	Literature review	Koob, GF; Volkow, ND	Neuropsychopharmacology	2010
14	3,134	Article	The PHQ-9: a new depression diagnostic and severity measure [32]	RCT	Kroenke, K; Spitzer, RL, Williams, JB	Psychiatric Annals	2002
15	3,068	Article	Screening for serious mental illness in the general population [33]	New measure	Kessler, RC; Barker, PR; Zaslavasky, AM	Archives of General Psychiatry	2003
16	3,057	Article	Acute and longer-term outcomes in depressed outpatients requiring one or several treatment steps: a STAR*D report [34]	RCT	Rush, AJ; Trivedi, MH; Fava, M	American Journal of Psychiatry	2006
17	2,946	Review	The World Mental Health (WMH) survey initiative version of the World Health Organization (WHO) Composite International Diagnostic Interview (CIDI) [35]	New measure	Kessler, RC; Ustun, TB	International journal of methods in psychiatric research	2004
18	2,883	Article	The prevalence and correlates of eating disorders in the national comorbidity survey replication [36]	Cross-sectional	Hudson, JI; Hiripi, E; Kessler, RC	Biological Psychiatry	2007
19	2,882	Review	Parkinson's disease: clinical features and diagnosis [37]		Jankovic, J	Journal of Neurology Neurosurgery and Psychiatry	2008
20	2,879	Article	A meta-analysis of cytokines in major depression [38]	Meta-analysis	Dowlati, Y; Herrmann, N; Lanctot, KL	Biological Psychiatry	2010
21	2,573	Article	NIMH Diagnostic Interview Schedule for Children Version IV (NIMH DISC-IV): description, differences from previous versions, and reliability of some common diagnoses [39]	New measure	Shaffer, D.; Fisher, P; Schwab-Stone, ME	Journal of the American Academy of Child and Adolescent Psychiatry	2000
22	2,559	Article	Multidisciplinary research priorities for the COVID-19 pandemic: a call for action for mental health science [40]	Literature review	Holmes, EA; O'Connor, RC ; Bullmore, E	Lancet Psychiatry	2020
23	2,548	Article	Evaluation of outcomes with citalopram for depression using measurement-based care in STAR*D: implications for clinical practice [41]	RCT	Trivedi, MH; Rush, AJ; Fava, M	American Journal of Psychiatry	2006
24	2,531	Article	Two formulas for computation of the area under the curve represent measures of total hormone concentration versus time-dependent change [42]	Statistical method	Pruessner, JC; Kirschbaum, C; Hellhammer, DH	Psychoneuroendocrinology	2003
25	2,517	Article	Prevalence and development of psychiatric disorders in childhood and adolescence [43]	Cross-sectional	Costello, E; Mustillo, S; Angold, A	Archives of General Psychiatry	2003
26	2,396	Article	The prevalence and correlates of adult ADHD in the United States: results from the National Comorbidity Survey Replication [44]	Cross-sectional	Kessler, RC; Adler, L; Zaslavsky, A	American Journal of Psychiatry	2006
27	2,380	Review	A neurotrophic model for stress-related mood disorders [45]	Literature review	Duman, RS; Monteggia, LM	Biological Psychiatry	2006
28	2,327	Review	Inflammation and its discontents: the role of cytokines in the pathophysiology of major depression [46]	Literature review	Miller, AH; Maletic, V; Raison, CL	Biological Psychiatry	2009
28	2,327	Article	The 16-item Quick Inventory of Depressive Symptomatology (QIDS), clinician rating (QIDS-C), and self-report (QIDS-SR): a psychometric evaluation in patients with chronic major depression [47]	New measure	Rush, AJ; Trivedi, MH; Keller, MB	Biological Psychiatry	2003
30	2,316	Review	Overweight, obesity, and depression a systematic review and meta-analysis of longitudinal studies [48]	Systematic review and meta-analysis	Luppino, FS; de Wit, LM; Zitman, FG	Archives of General Psychiatry	2010
31	2,298	Review	The enduring effects of abuse and related adverse experiences in childhood - a convergence of evidence from neurobiology and epidemiology [49]	Cross-sectional	Anda, RF; Felitti, VJ; Giles, WH	European Archives of Psychiatry and Clinical Neuroscience	2006
32	2,292	Article	The NimStim set of facial expressions: judgments from untrained research participants [50]	New dataset	Tottenham, N; Tanaka, JW; Nelson, C	Psychiatry Research	2009
33	2,269	Article	A randomized trial of an N-methyl-D-aspartate antagonist in treatment-resistant major depression [51]	RCT	Zarate, CA; Singh, JB; Manji, HK	Archives of General Psychiatry	2006
33	2,269	Article	Antidepressant effects of ketamine in depressed patients [52]	RCT	Berman, RM; Cappiello, A; Krystal, JH	Biological Psychiatry	2000
35	2,262	Article	Mindfulness-based stress reduction and health benefits - a meta-analysis [53]	meta-analysis	Grossman, P; Niemann, L; Walach, H	Journal of Psychosomatic Research	2004
36	2,253	Review	What is cognitive reserve? Theory and research application of the reserve concept [54]	Review	Stern, Y	Journal of the International Neuropsychological Society	2002
37	2,198	Review	Validity of the executive function theory of attention-deficit/hyperactivity disorder: a meta-analytic review [55]	Review and meta-analysis	Willcutt, EG; Doyle, AE; Pennington, BF	Biological Psychiatry	2005
37	2,198	Review	Neurocognitive deficits and functional outcome in schizophrenia: are we measuring the "right stuff"? [56]	Review and meta-analysis	Green, MF; Kern, RS; Mintz, J	Schizophrenia Bulletin	2000
39	2,196	Article	The size and burden of mental disorders and other disorders of the brain in Europe 2010 [57]	Cross-sectional	Wittchen, HU; Jacobi, F; Steinhausen, HC	European Neuropsychopharmacology	2011
40	2,175	Article	The Columbia-Suicide Severity Rating Scale: initial validity and internal consistency findings from three multisite studies With adolescents and adults [58]	New measure	Posner, K; Brown, GK; Mann, JJ	American Journal of Psychiatry	2011
41	2,142	Review	The reward circuit: linking primate anatomy and human imaging [59]	Review	Haber, SN; Knutson, B	Neuropsychopharmacology	2010
42	2,133	Review	Functional neuroimaging of anxiety: a meta-analysis of emotional processing in PTSD, social anxiety disorder, and specific phobia [60]	Meta-analysis	Etkin, A; Wager, TD	American Journal of Psychiatry	2007
43	2,131	Article	Testing mediational models with longitudinal data: questions and tips in the use of structural equation modeling [61]	Statistical method	Cole, DA; Maxwell, SE	Journal of Abnormal Psychology	2003
44	2,129	Article	The PHQ-8 as a measure of current depression in the general population [62]	Cross-sectional	Kroenke, K; Strine, TW; Mokdad, AH	Journal of Affective Disorders	2009
45	2,068	Review	The amygdala: vigilance and emotion [63]	Review	Davis, M; Whalen, PJ	Molecular Psychiatry	2001
46	2,062	Article	The Patient Health Questionnaire somatic, anxiety, and depressive symptom scales: a systematic review [64]	Systematic review	Kroenke, K; Spitzer, RL; Lowe, B	General Hospital Psychiatry	2010
47	2,055	Article	The psychological impact of the COVID-19 epidemic on college students in China [65]	Cross-sectional	Cao, WJ; Fang, ZW; Zheng, JZ	Psychiatry Research	2020
48	2,016	Article	Prevalence and co-occurrence of substance use disorders and independent mood and anxiety disorders - results from the national epidemiologic survey on alcohol and related conditions [66]	Cross-sectional	Grant, BF; Stinson, FS; Kaplan, K	Archives of General Psychiatry	2004
49	1,974	Article	The Posttraumatic Stress Disorder Checklist for DSM-5 (PCL-5): development and initial psychometric evaluation [67]	Cross-sectional	Blevins, CA; Weathers, FW; Domino, JL	Journal of Traumatic Stress	2015
50	1,964	Article	Psychiatric disorders in children with autism spectrum disorders: prevalence, comorbidity, and associated factors in a population-derived sample [68]	Cross-sectional	Simonoff, E; Pickles, A; Baird, G	Journal of the American Academy of Child and Adolescent Psychiatry	2008

Cross-sectional, new measure, literature review, and RCT publications were the most popular article types among the 50 highest-cited publications. For cross-sectional publications, the median number of citations was 2,396 (interquartile range (IQR): 2,129-3,183). For new-measure publications, the median number of citations was 2,559 (IQR: 2,450-3,573). For literature review publications, the median number of citations was 2,559 (IQR: 2,380-3,175). For RCT publications, the median number of citations was 2,548 (IQR: 2,269-3,057). The overall median across all publication types was 2,457 (IQR: 2,198-3,165). The three most popular journals within these top-cited publications included the American Journal of Psychiatry (eight publications), Biological Psychiatry (seven publications), and JAMA Psychiatry (formerly named Archives of General Psychiatry) (seven publications).

Top authors in psychiatry

The 50 most cited authors in psychiatry are listed in Table 2. The top five authors collectively had 445,740 citations and 3,635 publications. Of the 50 most-cited publications, female authors comprised 12 of the 50 (24%) first authors and 10 of the 50 (20%) senior authors. Of the 50 most cited authors in psychiatry, 11 (22%) were female authors. Notably, Ronald Kessler was the author of seven of the 50 publications. Of the 50 most cited authors in psychiatry, 27 (54%) authors were associated with a top 40 NIH-funded psychiatry program in the US. The top three university affiliations of top authors included Harvard University (five authors), Duke University (four authors), and Columbia University (three authors). All but one top author had a PhD/DrPH (23), MD (21), or both of these degrees (5).

**Table 2:** 50 top authors of the new millennium in psychiatry. NIH: National Institutes of Health.

Rank	Author last name, initial(s)	Times cited	Most cited article	Year published, most cited article	Avg. # most cited article was cited/year	Number of publications (y)	H-index (z)	Institution, country	At top-40 NIH funded program?	Highest post-graduate degree(s)
1	Kessler, RC	239,845	Lifetime prevalence and age-of-onset distributions' of DSM-IV disorders in the national comorbidity survey replication [19]	2005	829.3	1,134	229	Harvard University, United States	yes	PhD
2	Mokdad, AH	134,360	Global, regional, and national age-sex-specific mortality for 282 causes of death in 195 countries and territories, 1980-2017: a systematic analysis for the global burden of disease study 2017 [69]	2018	1312	626	135	University of Washington, United States	no	PhD
3	Volkow, ND	96,706	Neurocircuitry of addiction [31]	2010	234.9	1245	150	National Institutes of Health, United States	no	MD
4	Kroenke, K	96,081	The PHQ-9 - validity of a brief depression severity measure [70]	2001	941	387	101	Indiana University, United States	no	MD
5	Koob, GF	92,437	Neurocircuitry of addiction [31]	2001	234.9	1037	156	National Institutes of Health, United States	yes	PhD
6	Wittchen, HU	77,406	Lifetime and 12-month prevalence of DSM-III-R psychiatric disorders in the United States - results from the National-Comorbidity-Survey [71]	1994	313.4	967	127	Ludwig Maximilian University of Munich, Germany	no	PhD
7	Williams, JBW	70,938	The PHQ-9 - validity of a brief depression severity measure [70]	2001	941	243	53	Columbia University, United States	yes	PhD
8	Stern, Y	67,024	Toward defining the preclinical stages of Alzheimer's disease: recommendations from the National Institute on Aging-Alzheimer's Association workgroups on diagnostic guidelines for Alzheimer's disease [72]	2011	339.8	527	128	Columbia University, United States	yes	PhD
9	Rush, AJ	66,835	The epidemiology of major depressive disorder - Results from the National Comorbidity Survey Replication (NCS-R). [73]	2003	274	588	119	Duke University, United States	yes	MD
10	Grant, BF	62,628	A comparative risk assessment of burden of disease and injury attributable to 67 risk factors and risk factor clusters in 21 regions, 1990-2010: a systematic analysis for the Global Burden of Disease Study 2010 [74]	2012	634.8	354	108	National Institutes of Health, United States	no	PhD
11	Duman, RS	59,793	Requirement of hippocampal neurogenesis for the behavioral effects of antidepressants [75]	2003	157.2	449	120	Yale University, United States	yes	PhD
12	Mann, JJ	57,895	The Columbia-Suicide Severity Rating Scale: initial validity and internal consistency findings from three multisite studies with adolescents and adults [58]	2011	184.2	997	121	Columbia University, United States	yes	MD
13	Fava, M	56,676	Acute and longer-term outcomes in depressed outpatients requiring one or several treatment steps: A STAR*D report [34]	2006	179.6	1071	110	Harvard University, United States	yes	MD
14	Walters, EE	50,856	Lifetime prevalence and age-of-onset distributions' of DSM-IV disorders in the national comorbidity survey replication [19]	2005	829.3	61	44	University of Southern California, United States	yes	MS
15	Krystal, JH	49,167	Subanesthetic effects of the noncompetitive NMDA antagonist, ketamine, in humans - psychotomimetic, perceptual, cognitive, and neuroendocrine responses [76]	1994	84.87	754	117	Yale University, United States	yes	MD
16	Wager, TD	48,804	The unity and diversity of executive functions and their contributions to complex "frontal lobe" tasks: a latent variable analysis [77]	2000	369.1	357	84	Dartmouth College, Unites States	yes	PhD
17	Zaslavasky, AM	46,964	Short screening scales to monitor population prevalences and trends in non-specific psychological distress [21]	2002	268.8	431	88	Harvard University, United States	yes	PhD
18	Baron-Cohen, S	44,994	Autism [78]	2014	131.4	244	96	University of Cambridge, England	no	PhD
19	Trivedi, M H	44,882	Acute and longer-term outcomes in depressed outpatients requiring one or several treatment steps: A STAR*D report [34]	2006	179.6	807	98	The University of Texas Southwestern Medical Center, United States	yes	MD
20	Insel T	44,443	The Genotype-Tissue Expression (GTEx) project [79]	2013	396.6	347	100	National Institutes of Health, United States	no	MD
21	Merikangas, KR	39,841	The epidemiology of major depressive disorder - Results from the National Comorbidity Survey Replication (NCS-R) [73]	2003	274	381	83	National Institutes of Health, United States	no	PhD
22	Löwe, B	35,373	A brief measure for assessing generalized anxiety disorder - The GAD-7 [80]	2006	719.7	344	55	University of Heidelberg, Germany	no	MD
23	Anda, RF	35,232	Relationship of childhood abuse and household dysfunction to many of the leading causes of death in adults - the adverse childhood experiences (ACE) study [81]	1998	350	139	69	Centers for Disease Control and Prevention, United States	no	MD
24	Costello, E	35,100	Prevalence and development of psychiatric disorders in childhood and adolescence [43]	2003	122.5	211	97	Duke University, United States	yes	PhD
25	Giles, WH	31,927	Prevalence of the metabolic syndrome among US adults - findings from the Third National Health and Nutrition Examination Survey [82]	2002	221.3	237	77	University of Illinois at Chicago, United States	no	MD
26	Wang, PS	30,936	The epidemiology of major depressive disorder - Results from the National Comorbidity Survey Replication (NCS-R) [73]	2003	274	75	51	Harvard University, United States	yes	MD, DrPH
27	Zarate, CA	30,380	A randomized trial of an N-methyl-D-aspartate antagonist in treatment-resistant major depression [51]	2006	131.6	633	93	National Institutes of Health, United States	no	MD
28	Ustun, TB	30,379	The World Mental Health (WMH) Survey Initiative version of the World Health Organization (WHO) Composite International Diagnostic Interview (CIDI) [35]	2004	152.4	105	52	Koc University, Türkiye	no	MD
29	Miller, AH	29,560	Inflammation and its discontents: the role of cytokines in the pathophysiology of major depression [46]	2009	162.3	361	74	Emory University, United States	yes	MD
30	Polanczyk, G	29,347	Disability-adjusted life years (DALYs) for 291 diseases and injuries in 21 regions, 1990-2010: a systematic analysis for the Global Burden of Disease Study 2010 [83]	2012	471.7	173	42	University of São Paulo Medical School, Brazil	no	MD, PhD
31	Manji, HK	27,330	A randomized trial of an N-methyl-D-aspartate antagonist in treatment-resistant major depression [51]	2006	131.6	412	90	Oxford University, England	no	MD
32	Hellhammer, DH	26,383	The Trier Social Stress Test- a tool for investigating psychobiological stress responses in a laboratory setting [84]	1993	128.4	232	65	University of Trier, Germany	no	PhD
33	Davidson, JR	26,149	Development of a new resilience scale: The Connor-Davidson Resilience scale (CD-RISC) [23]	2003	216.3	340	74	Duke University, United States	yes	MD
34	Pruessner, JC	24,124	Two formulas for computation of the area under the curve represent measures of total hormone concentration versus time-dependent change [42]	2003	124.8	360	76	McGill University, Canada	no	PhD
35	Mintz, J	23,861	Neurocognitive deficits and functional outcome in schizophrenia: are we measuring the "right stuff"? [56]	2000	93.33	345	82	University of California, Los Angeles, United States	yes	PhD
36	Angold, A	23,654	Prevalence and development of psychiatric disorders in childhood and adolescence [43]	2003	122.5	125	71	Duke University, United States	yes	MD
37	Keller, MB	23,378	The 16-item Quick Inventory of Depressive Symptomatology (QIDS), clinician rating (QIDS-C), and self-report (QIDS-SR): a psychometric evaluation in patients with chronic major depression [47]	2003	116.3	346	73	Brown University, United States	no	MD
38	Steinhausen, HC	23,131	The size and burden of mental disorders and other disorders of the brain in Europe 2010 [57]	2011	176.5	342	73	University of Southern Denmark, Denmark	no	MD, PhD
39	Knutson, B	22,406	The reward circuit: linking primate anatomy and human imaging [59]	2010	162.1	154	65	Stanford University, United States	yes	PhD
40	Walker, EF	21,623	Diagnostic and statistical manual of mental disorders [20]	2011	5535	378	55	Emory University, United States	yes	PhD
41	Haber, SN	21,282	The reward circuit: linking primate anatomy and human imaging [59]	2010	162.1	211	72	University of Rochester, United States	yes	PhD
42	Nelson, C	21,065	The NimStim set of facial expressions: judgments from untrained research participants [50]	2009	160	317	74	Harvard University, United States	yes	PhD
43	Baird, G	20,939	Psychiatric disorders in children with autism spectrum disorders: prevalence, comorbidity, and associated factors in a population-derived sample [68]	2008	127.8	171	66	Guy's and St Thomas'​ NHS Foundation Trust, United States	no	MD
44	Rohde, LA	18,699	The worldwide prevalence of ADHD: a systematic review and metaregression analysis [29]	2007	197.6	411	54	The Federal University of Rio Grande do Sul, Brazil	no	MD, PhD
45	Goodman R.	17,963	Psychometric properties of the strengths and difficulties questionnaire [25]	2001	183.2	124	58	Kings College London, England	no	PhD
46	Simonoff, E	17,796	Psychiatric disorders in children with autism spectrum disorders: prevalence, comorbidity, and associated factors in a population-derived sample [68]	2008	127.8	177	64	Kings College London, England	no	MD
47	Whalen, PJ	17,684	The amygdala: vigilance and emotion [63]	2001	91.96	100	48	University of Wisconsin-Madison, United States	yes	PhD
48	Etkin, A	17,321	Functional neuroimaging of anxiety: a meta-analysis of emotional processing in PTSD, social anxiety disorder, and specific phobia [60]	2007	129.7	127	49	Stanford University, United States	yes	MD, PhD
49	Monteggia, LM	16,625	A neurotrophic model for stress-related mood disorders [45]	2006	135.7	139	49	Vanderbilt University, United States	yes	PhD
50	Raison, CL	16,569	Inflammation and its discontents: the role of cytokines in the pathophysiology of major depression [46]	2009	162.3	125	47	University of Wisconsin-Madison, United States	yes	MD

Discussion of findings

This study used the Web of Science (WOS) database of articles published in psychiatric journals from 01/01/2000-09/18/2022 to identify the 50 most cited publications as well as the 50 most cited authors for a bibliometric analysis. Within the 50 most cited psychiatric journal publications of the new millennium, the analysis found that, first, these publications were cited between 1,964 and 15,001 times. Second, the most popular types of highly cited articles were cross-sectional studies, new measures, literature reviews, and randomized controlled trials. Third, the journal with the most top 50 highest-cited publications was the American Journal of Psychiatry, with eight out of the top 50 publications.

Our authorship analysis revealed, first, that female authors remain underrepresented in recent advances in psychiatry, comprising 12 of the 50 first authors of top psychiatry articles and 10 of the 50 senior authors of top psychiatry articles. Within the 50 top authors, just 11 (22%) were female authors. This percentage is similar to the 23% of psychiatry department chairs who were female in a recent analysis of women in academic psychiatry, despite women accounting for 34% of full professors in the field [85]. Second, our analysis showed that 27 (54%) top-50 authors were affiliated with a top 40 NIH-funded psychiatry program in the US, with an additional six (12%) authors affiliated with the NIH itself. Thus, approximately two-thirds of top authors in psychiatry across the world work within the NIH or one of the top 40 US psychiatry departments to which it grants funding. A third of top authors in psychiatry were affiliated with just four US institutions: 36% of top authors were affiliated with the NIH, Harvard University, Duke University, or Columbia University. Third, this analysis shows that MDs and PhDs appear to contribute to top-cited articles in psychiatry relatively equally; from this analysis, it appears that holding an MD or a PhD is nearly universal among top authors in the field, but those with either degree can reach the top levels of impact in the field.

Many bibliometric studies have concentrated on specific subfields of psychiatry rather than the general field; one salient exception was a bibliometric analysis conducted by Mazhari in 2013 [15]. There is a paucity of studies that provide a broad survey of the recent psychiatric advances of the past two decades. Mazhari’s study focused on the top 100 publications and considered publications between 1957 and 2005 [15]. This study includes the characteristics of the most-cited researchers across psychiatry in addition to the top publications, an additional dimension that adds to prior field-wide knowledge of top articles. Our criteria for inclusion as a “top author” presents a more nuanced look at leading authorship compared to measures such as H-index and provides a less ephemeral look at productive contributions to the field than resources such as the Blue Ridge Institute’s yearly characterization of top grant-getters in psychiatry [18]. Thus, this study attempts to fill the gap in current literature by highlighting recent advances and those at the forefront of psychiatry in a manner that captures recent and lasting literary impact. Furthermore, it examines an uncharacterized recent period in the field, starting in 2000.

This study had several notable limitations. First, when the publications found in the WOS database were cross-verified with PubMed, this revealed that the article “Diagnostic and Statistical Manual of Mental Disorders” was erroneously reported to be cited more than it actually had been in the WOS database. Other limitations of this data source and our search criteria are the limited inclusion of relevant articles in psychiatry published in journals nonspecific to psychiatry, including the Clinical Antipsychotic Trials of Intervention Effectiveness (CATIE) trial and genome-wide association studies showing relevance for bipolar I disorder [86,87]. Other notable exclusions include articles published by NEJM, JAMA, and JAMA Internal Medicine pertaining to opioid- and cannabis-related areas of study of interest to both psychiatrists and general medicine. Finally, this study did not investigate the temporal aspect of the top publications or authors; thus, some publications could potentially be cited more frequently simply due to their earlier date of publication than because of their relative contribution to psychiatry.

## Conclusions

This study is one of the few to report top authors and articles in psychiatry for those interested in a broad overview of the field. More specifically, we highlight the characteristics of the most-cited publications and authors in psychiatry, which shows both types of articles and the authors who end up receiving the largest number of citations. Understanding these recent top authors and their highly cited articles can serve as a useful starting point for anyone wishing to obtain a broad education in the recent history of psychiatry, as well as those one day hoping to become top authors in the field themselves.
